# “Adolescents do not only require ARVs and adherence counseling”: A qualitative investigation of health care provider experiences with an HIV youth peer mentoring program in Ndola, Zambia

**DOI:** 10.1371/journal.pone.0252349

**Published:** 2021-06-09

**Authors:** Elizabeth A. Abrams, Virginia M. Burke, Katherine G. Merrill, Christiana Frimpong, Sam Miti, Jonathan K. Mwansa, Julie A. Denison

**Affiliations:** 1 Department of International Health, Johns Hopkins Bloomberg School of Public Health, Baltimore, Maryland, United States of America; 2 Arthur Davison Children’s Hospital, Ndola, Zambia; Population Council, INDIA

## Abstract

**Introduction:**

Adolescents and young adults (AYAs) living with HIV face unique challenges and have poorer health outcomes than adults with HIV. Project YES! was a youth-led initiative to promote HIV self-management and reduce stigma among AYAs in four Ndola, Zambia clinics. Clinic health care providers (HCPs) were involved in multiple intervention aspects, including serving as expert resources during AYA and caregiver group meetings, facilitating resistance test-based AYA antiretroviral drug changes, meeting with participants referred through a safety protocol, and guiding a subset of participants’ physical transition from pediatric to adult clinic settings. This study aimed to understand HCP insights on facilitators and barriers to implementing Project YES! and scaling up a clinic-based, youth-focused program.

**Methods:**

A trained interviewer conducted ten in-depth interviews with participating HCPs from November–December 2018 and analyzed data, identifying key themes. These themes were examined in terms of two implementation science outcomes–acceptability and feasibility–to inform scalability.

**Results:**

HCPs found peer mentoring valuable for AYAs with HIV and the bimonthly caregiver meetings beneficial to AYA caregivers. HCPs voiced a desire for more involvement in specific processes related to patient clinical care, such as drug changes. HCPs’ experiences with the study safety protocol, including referrals for youth experiences of violence, shifted their views of AYAs and informed their understanding of key issues youth face. Considering this, many HCPs requested more resources to support AYAs’ varied needs. HCPs noted limited time and clinic space as implementation barriers but felt the program was valuable overall.

**Conclusions:**

HCPs concluded youth peer mentoring was highly acceptable and feasible, supporting scale-up of youth-led interventions addressing the multi-faceted needs of AYAs living with HIV. Continued provider involvement in resistance test-based antiretroviral drug changes, considered in the context of health system and clinic policy, would enhance long-term success of the program at scale.

## Introduction

Adolescents and young adults (AYAs) living with HIV have lower adherence levels, less viral suppression [1–5], and higher mortality rates [6] than adults with HIV. Clinic-based approaches can support AYAs during regular HIV care visits. However, clinic health care providers (HCPs) in sub-Saharan Africa often work within overburdened health systems [7–9] and may lack resources to address issues AYAs face that may impact HIV outcomes, including complex, developmentally-based physical, social, and cognitive changes [10–13].

The HIV field recognizes AYAs’ important roles combatting the HIV epidemic, with a worldwide call for youth engagement and youth-led HIV prevention and support initiatives [14–17]. In response, a randomized controlled trial (RCT), Project YES! (Youth Engaging for Success!) tested the impact of a clinic-based HIV youth peer mentoring program on AYA viral suppression in four Ndola, Zambia HIV clinics (one pediatric, three adult). Eight AYAs living with HIV were trained and hired to mentor other AYAs with HIV with a goal of strengthening self-management and increasing viral suppression. Impact data found intervention arm participants had significantly reduced internalized stigma (e.g., feeling of worthlessness) relative to comparison participants [interaction term OR: 0.39, 95% CI: 0.21, 0.73] [18]. Stratified analysis found that among pediatric clinic participants, there was significant increase in viral suppression in the intervention arm relative to the comparison arm [interaction term OR: 4.66, 95% CI: 1.84, 11.78] [18]. Based on these data, CDC included Project YES! in the Compendium of Evidence-Based Interventions and Best Practices for HIV Prevention [19].

When considering these data and Project YES! scale-up, it is vital to examine experiences of HCPs who oversaw implementation in their clinics. Although HCPs play a critical role in clinic-based programs, their experiences as implementing partners are rarely reported. Implementation science offers tools for examining these perspectives using frameworks and approaches for scaling up successful interventions [20,21]. This paper presents findings from in-depth interviews with HCPs to understand facilitators and barriers to Project YES!, and then examines key themes in terms of two implementation science outcomes–feasibility and acceptability–to inform Project YES! scale-up [22].

## Methods

### Project YES! intervention: Study background and procedures

Project YES! hired 21-26-year-olds living with HIV for capacity-building to become trained youth peer mentors (YPMs), working as lay staff members at four Ndola, Zambia clinics. Treatment-experienced AYAs aged 15–24 years were consecutively recruited and enrolled before being randomly assigned to intervention or comparison arms for a six-month intervention. HCPs at each clinic supervised YPMs, described elsewhere [18]. Table 1 describes the five Project YES! intervention components and HCP roles in implementation. HCPs were engaged before and throughout implementation (e.g., involvement in YPM selection and management) and worked with study team members to monitor and adjust the program. HCPs were compensated for their roles.

**Table 1:** Project YES! components and HCP^†^ roles in component implementation.

Intervention Component	Component Description	HCP Role Implementing Component
**One-on-one and Group Meetings with YPMs^‡^**	• YPMs were trained and employed by Project YES! as clinic staff. • YPMs held one orientation meeting with AYA^§^ clients and HCPs, six monthly one-on-one meetings (AYAs and YPMs only), and six-monthly group meetings (AYAs with YPMs as facilitators) for intervention arm participants.	• Led and facilitated orientation meetings with YPMs, AYA clients, and caregivers (if invited by AYAs); met with invited caregivers separately • Identified clinic space for meetings • Answered AYAs’ questions beyond scope of YPM knowledge
**Caregiver Group Meetings**	• Caregivers, if invited by AYAs, could choose to attend 3 bimonthly caregiver group meetings.	• Facilitated caregiver group meetings using Project YES! group meeting outlines
**ART^¶^ Drug Change**	• AYAs with baseline viral load failure were tested for drug resistance. • The study pediatrician reviewed test results and patient files and recommended ART drug changes as necessary.	• Facilitated patients’ drug changes
**Physical Transition from Pediatric to Adult Clinic**	• Intervention participants in the pediatric clinic who were medically and psychosocially eligible were invited to a transition tour of the adult clinic (with YPMs and HCPs). • AYAs were given a letter for their caregivers explaining the transition. • If they accepted transition, AYA started the intervention and clinical care at the adult clinic.	• Pediatric clinic HCPs assessed AYAs’ transition eligibility and were present at the transition tour of the adult facility. • Pediatric clinic HCPs attended initial adult clinic visits with AYA clients (not systematically applied). • Adult clinic HCPs facilitated the transition tour and welcomed new AYAs to the clinic.
**Safety Protocol**	• AYAs who reported experiences of severe violence, suicide ideation, or other clinical and social concerns on study surveys or during the program were referred to designated HCPs.	• HCPs met with AYAs and further referred them as needed, handling cases according to clinical practice, local policy, and Zambian law.

† Health care provider, ‡ Youth peer mentor, § Adolescent and young adult, ¶ Antiretroviral therapy.

### Qualitative data collection and analysis

In-depth interviews were conducted with Project YES! HCPs from November–December 2018 after data collection for the primary RCT impact analyses was completed. HCPs were selected based on their participation in the program. All HCPs who served a key role during the study period and were still working at the clinics at the time of interview were approached in person to request an interview, and none refused. Of ten interviewed, one was a lay counselor who assisted tracing AYAs in the community, one a pharmacist who dispensed antiretroviral therapy (ART), one a pediatrician, and seven with nursing training, including one trained in mental health. The pediatrician was also a study co-investigator who was selected for interviewing based on Project YES! clinical involvement, including reviewing resistance test results and recommending ART regimen changes.

A female graduate student (EA) who worked with the Project YES! team and had extensive qualitative research training conducted all interviews in private clinic or hospital settings, each lasting 1.5–2 hours. Eight interviews happened once, and for two informants the interview was broken into two parts due to HCP time constraints. Interview guides, developed and reviewed by the research team, drew upon the implementation science framework described below to understand implementation challenges and successes to inform scale up of effective services (S1 Interview guide). Questions focused on HCPs’ experiences with YPMs, caregiver group sessions, transitioning AYAs to adult care, ART regimen changes, and safety protocol implementation, as well as overall experiences working with HIV-positive AYAs. Questions also asked about HCPs’ professional backgrounds and clinic roles. Social desirability bias was minimized by interviewer’s presence on site over time and trust-building with HCPs.

Detailed memos were made after each interview. Interviews were also audio-recorded, transcribed, spot-checked for accuracy, and summarized by the primary author (EA). Transcripts were imported to NVivo 12, a qualitative software package, for data management and organization. A codebook was developed and applied by the interviewer using an inductive approach; while initial codes were derived from interview questions, additional codes emerged from the data during analysis. Code families included: Background, Overall Program Experiences, Youth Peer Mentors, Caregivers, Transition, Youth Self-management, Drug Change, Referrals, and Rich Quotes, and 53 unique codes fell under these categories. Themes were identified with research team input and saturation achieved on key themes presented. HCPs participated in dissemination activities in which data were presented and discussed.

### Analysis implementation framework

Implementation science offers many frameworks for successful intervention scale-up. One model, published by Proctor et al., details a “Taxonomy of implementation outcomes,” with eight outcomes [22]. Among these are acceptability and feasibility [22]. Analyzed at the individual level, acceptability and feasibility are relevant outcomes when considering Project YES! scale-up due to the value of buy-in and implementation ease from key stakeholders, including HCPs. Acceptability encompasses satisfaction or relative advantage offered by an intervention component. Feasibility relates to a component’s practicality or fit within a context. We present five key themes from HCP IDIs before using them to discuss the intervention’s overall acceptability and feasibility from HCPs’ perspectives.

### Ethical and consent considerations

This study was reviewed and approved by the Eres Converge Institutional Review Board in Zambia; the Ministry of Health–Zambia, through the National Health Research Authority; and the Johns Hopkins Bloomberg School of Public Health Institutional Review Board in the United States. Written informed consent including information about research purpose was obtained from HCPs prior to interviews.

## Results

Five themes emerged from HCP interview data regarding peer mentors’ overall value, HCP involvement in clinical processes and decision making, caregiver roles, HCP experiences and transformation through safety protocol implementation, and challenges with time and space.

### Theme 1: Peer mentoring benefitted AYAs

HCPs recognized YPMs’ unique abilities to connect with AYAs living with HIV, noting YPMs were more effective and trusted information messengers than adult clinic providers because YPMs–also living with HIV–had encountered similar struggles as AYAs. One HCP explained YPMs’ distinct value:

“[The YPMs and I] complemented each other. They were everything I’m not. I’m not an adolescent…I’m not going through what they’re going through…So, most of the things that I couldn’t do for this peer, they were able to…”

HCPs expressed confidence in YPMs’ leadership of one-on-one and group meetings, noting that YPMs were well-trained, needed little supervision, and recognized boundaries. HCPs saw YPMs as assets within general clinic environments. When not meeting with Project YES! clients, YPMs helped with other tasks like pulling medical charts, filing results, and showing new patients around.

### Theme 2: HCPs wanted more involvement in project YES! decisions and processes related to patients’ clinical care

HCPs identified two Project YES! components where they wanted more agency and better communication: ART drug changes due to study-initiated resistance testing and physical transition of eligible AYAs from the pediatric to adult clinic.

#### HCP involvement in ART drug change

Some HCPs noted challenges with the drug change process, describing two main reasons for reluctance to change some participants’ drugs despite the study physician’s resistance test-informed recommendation. First, while some HCPs felt the process was smooth, others emphasized it could be enhanced by moving more slowly. As one HCP said, AYAs should be “religiously adherent” to current regimens before changing ART drugs. They felt that, regardless of AYAs’ ART resistance, establishing strong adherence practices before changing medications would lower risk of developing resistance to the new drug. HCPs described this “slower” process as their typical procedure for changing ART drugs.

Second, multiple HCPs indicated a desire for stronger communication across clinic staff during the drug change process. HCPs felt the physician interpreting resistance test results should have communicated more frequently with other HCP(s) involved in participants’ medical care when considering drug changes. One HCP explained the need for stronger communication that engaged more health care team members:

“You know, when you are doing drug change, you need to work as a team…the clinician should be there…the nurses, the pharmacist, as well as even the counselor.”

#### HCP involvement in physically transitioning AYAs to adult care setting

Seven of the ten HCPs interviewed discussed AYA transition from pediatric to adult clinic settings, including six from the two clinics involved in the Project YES! transition process. HCPs described multiple benefits of this transition. First, physical transitions made room at the pediatric clinic for new patients. Second, before Project YES!, AYAs typically only physically transferred from the pediatric to an adult clinic when they had a medical problem and needed admission to the adult hospital, because the children’s hospital does not admit anyone over age 15. Pediatric clinic HCPs explained how AYAs who were transferred to the adult clinic by emergent admission to the adult hospital either stopped going to the adult HIV clinic altogether or returned to the pediatric ART clinic once better because the adult clinic was, in AYAs’ words, “not very welcoming.” HCPs therefore felt that Project YES! offered an improved model by facilitating a planned transition process that supported healthy and willing patients moving from pediatric to adult care. HCPs appreciated several transition process characteristics, including the adult clinic group transition tour that promoted communication among providers, YPM presence and mentoring at the adult clinic, and the plan to have pediatric clinic HCPs attend the first adult clinic appointments with transitioned youth (although this did not occur systematically for all transitioned youth).

One HCP noted communication between pediatric and adult providers is critical to successful transitions, and communication with patients during this process would be enhanced by having the patients present when the HCP evaluates their transition readiness.

“Maybe next time we do it in presence of the client…I look at the file, go through it, look at all the labs, I’m comfortable with them, then now we speak, talk about transitioning…”

### Theme 3: HCPs valued caregiver meetings and encouraged greater caregiver involvement

As reported in the Project YES! primary paper, 59 (47%) of 126 intervention arm participants with results analyzed at midline had a caregiver attend at least one Project YES! session [18]. Several HCPs reported caregivers were engaged in group meetings and asked many questions about HIV myths and misconceptions, herbal medicines, and sexual and reproductive health. HCPs felt addressing sensitive topics during group meetings helped caregivers learn to discuss the same challenging issues with their AYAs. One HCP shared how sex came to be an appreciated topic during caregiver meetings:

“So we actually guided them on how to go about [discussing sensitive topics like sex], how to start the conversations with the young ones, and when the young ones come up with any question concerning that, we were telling them never to shut them up but to help in a way. So, when they [caregivers] learnt that even these young ones are learning about sex [in the youth group meetings] they were happy.”

While all AYA participants were aware of their HIV status prior to enrolling in Project YES!, HCPs also noted that caregivers particularly valued discussing their past experiences with disclosing to their youth that the young person was living with HIV. Caregivers often felt residual guilt from these experiences and suffered communication barriers with their AYAs depending on how disclosure had occurred. Group meetings, according to HCPs, gave caregivers space to work through disclosure-related issues in a previously unavailable format.

#### Caregiver involvement in ART drug change

HCPs agreed caregiver involvement in the drug change process helped encourage AYA adherence to their new ART regimens. One HCP felt Project YES! needed to lay out specific steps for further caregiver involvement in drug changes, even among youth 18 and older who are viewed as adults:

“because there’s no way you can change somebody’s son or daughter the regimen without the knowledge of their parents. So we needed to inform and talk to the parents first…”

#### Caregiver involvement in physically transitioning AYAs to adult care setting

Most HCPs involved in the transition process advocated for further engaging caregivers. HCPs noted multiple occasions in which AYAs indicated a willingness to transition, but caregivers did not allow it. One HCP described a caregiver’s response about transitioning her child:

“The caregiver…was not happy about it. She said: ‘If you had involved me from the word go, I think, maybe I would have understood it better. But because I don’t understand it better…I know you are explaining now, it’s too late. I can’t release my daughter.’”

HCPs made three suggestions to further involve caregivers and improve the transition experience. First, while the Project sent letters to caregivers notifying them of an AYA’s transition and requesting permission from minors’ caregivers, some HCPs suggested even greater caregiver involvement–specifically, an in-person conversation between the HCP, AYA, and caregiver about whether an AYA is ready to transition, so caregivers can give input. Second, one HCP recommended caregivers be transitioned along with their AYAs, when applicable, as some adult family members still receive ART care from the pediatric clinic as part of a family program. The associated logistical barriers of having members of the same family receiving care from multiple clinics, as well as caregivers’ own lack of familiarity with the adult clinic, make it unlikely that caregivers would allow their AYA to move to the adult clinic before themselves. Third, HCPs suggested a transition tour specifically for caregivers where they could ask questions and meet other caregivers considering transitioning their AYAs. HCPs explained caregivers often have their own myths about the adult clinic; they may have heard it is unfriendly, worry about stigma associated with being seen there, or have had negative experiences themselves. A transition tour for caregivers could help alleviate these concerns.

### Theme 4: The safety protocol referral process helped HCPs recognize the intersectional challenges AYAs living with HIV face, and HCPs desired expanded capacity to support AYAs in overcoming these barriers

HCPs felt the safety protocol (Table 1) was an overall Project YES! strength. One HCP shared this example of how supporting a client through the safety protocol led to changes in both the AYA and HCP:

“She had been abused…And the first time she disclosed it was to the Project YES! research staff that were first screening them…Then she opened up…So now after we did all those sessions, then I saw this kid now finally coming alive, realizing that she’s taking some of the weight off the shoulders, even when I did the referral to the…trauma counselor. Ah! Now she picked up. So the courage came back. So it was an interesting situation where you could really feel you have done something to help someone when you see them change even the way they like approach issues now.”

By talking with referred AYAs, HCPs said they gained better recognition of varied challenges HIV-positive AYAs encounter. HCPs were moved by AYAs’ stories and noted this would change how they engage with AYAs. One HCP described how YPMs helped them recognize challenges and needs of HIV-positive AYAs:

“Some of the patients that I thought had no problems, had no issues, I’ve just realized that you don’t take it for granted…So the peer mentors have actually done a very good job because they ask so many questions and they are the ones that have opened my eyes to know that these adolescents do not only require ARVs and adherence counseling. They need to be asked a lot of questions so that you get to understand where they are, how they’re feeling, what problems they’re going through.”

HCPs noted connections between AYAs’ challenges and ART adherence, as illustrated by this story of an AYA who stopped taking ART due to verbal abuse:

“I had one encounter…with one guy who used to say, “You know, some statements that some people make, something like, ‘Oh, because you are reactive, you are just a moving coffin…’.” Can you imagine that running through your mind from time and time again? There’s one time it led him to default, he stopped. Then after some time…they learnt that he had stopped because his viral load has gone up. Then they initiated him on second line. So I think that’s how it can affect the adherence…So you feel the sense of worth is not there…even if you take [ART drugs] or you don’t take, it doesn’t matter.”

HCPs expressed desire for increased capacity to meet AYAs’ complex needs. They felt they had limited time to meet with AYAs and adequately address issues at the clinic, and lack of knowledge of community resources for AYA referral.

### Theme 5: Limited HCP time and clinic space posed implementation challenges

HCPs had limited time for project activities. As critical players in an overburdened health system, HCPs expressed that drawing blood, leading participant orientation meetings, meeting with AYAs referred through the safety protocol, and facilitating caregiver group meetings were difficult to balance with other responsibilities and posed implementation barriers. HCPs proposed delegating some tasks to others, like having psychosocial counselors who typically focus on adherence counseling manage safety protocol referrals.

Finally, most HCPs acknowledged the challenge of limited clinic space. Finding comfortable, confidential spaces for YPMs to meet with clients was an ongoing issue solved on case-by-case bases. One HCP expressed not only the concern about space, but a sense that there is nothing HCPs can do to address it: “So as for space, it’s a big challenge. It’s beyond me.”

## Discussion

HCPs found youth peer mentoring highly acceptable and, even with some implementation challenges, highly feasible. HCPs valued the unique role YPMs fulfilled and clearly described the advantage of having young people on staff who could connect with AYA clients through shared experiences. HCPs felt comfortable and satisfied with YPMs and their work, affirming the acceptability of Project YES! peer mentoring among HCPs. In terms of feasibility, YPMs fit into the clinic environment well and were seen as professional assets to providing youth-friendly HIV services. As the value of adolescent peer-led programs is being recognized [23–25], their scalability depends in part on the contexts in which programs will operate. Given that Project YES! was clinic-based and required close collaboration between peers and HCPs, HCPs’ affirmation of the peer mentoring model’s acceptability and feasibility is an indicator supporting program scalability.

HCPs also found the Project YES! guided transition process and safety protocol highly acceptable. The safety protocol provided space for HCPs to discuss issues that could impact HIV-related behaviors but are not typically addressed in HIV clinics with AYA clients, including AYAs’ frequent experiences of physical, sexual, and psychological violence [26–28]. These interactions changed how HCPs viewed and interacted with AYAs in their clinics–meaningful shifts that reveal how the safety protocol was highly valued and acceptable for HCPs. Previous literature has identified that patient-provider relationships influence ART retention among adolescents [9,29,30]. However, this kind of reflexivity, in which HCPs have space to reflect on how their own perceptions of patients have evolved and how these changes have influenced practice, is not often examined in intervention research and should continue to be prioritized to build workforce capacity. Overall, HCP support of the safety protocol suggests they would value this component in future work with AYAs living with HIV. Further, the HCPs’ emphasis on increasing caregiver engagement in the transition and treatment processes should be incorporated as the program is scaled up. Feasibility challenges such as limited HCP time and clinic space were ongoing realities throughout implementation, aligning with existing studies identifying similar barriers from the youth perspective [30,31]. Future programs should thoroughly account for these potential barriers from the beginning.

Our results also highlight the structural and contextual factors necessary to consider for scale up and the need to consistently engage HCPs in clinical project components from the start. During the study, HCPs operated under 2018 Zambia Consolidated HIV Guidelines for HIV Prevention and Treatment [32]. These stipulated that ART drug changes follow two viral load tests of ≥1000 copies/mL in a 3-month period, with adherence support between tests [32]. Resistance genotype testing was only recommended for switching patients to third line drug regimens that were only available in two public hospitals. Project YES! study procedures relied on the results of only one viral load test; if a viral load was ≥1000 copies/mL, a resistance test was done to determine whether participants were on an effective HIV drug regimen. The study’s data on resistance testing was among the evidence considered in development of Zambia’s revised guidelines, published in 2020 [33]. Resistance testing is now recommended for all patients switching to a second line drug after two viral load tests indicate viral failure, and adherence issues are addressed [34]. Future iterations of this intervention should include conformity to prevailing HIV drug resistance testing protocols and more transparent communication and orientation with HCPs to incorporate appropriate resistance testing practices [34]. Deeper engagement of HCPs–and caregivers–would increase acceptability of the drug change process from the HCP perspective and enhance the intervention’s sustainability and scalability within broader health system policy.

One study limitation is not all HCPs interviewed were present throughout the whole intervention, and the role of each HCP differed by the clinic where they were based (e.g., most adult clinic HCPs were not involved in patient transition from the pediatric to adult hospital clinic), so exposure to some intervention components may have been low for some HCPs. Future work is needed to understand the impact of new resistance testing guidelines on implementation and ways reported changes in HCP practice after meeting with youth through the safety protocol have been sustained.

## Conclusions

Our work highlights three messages regarding Project YES! and potential scale-up of this acceptable and feasible peer mentoring intervention. First, it is key to build on successful elements that HCPs found most acceptable and feasible: use of youth peer mentoring, the guided transition process, and the safety protocol. Second, leveraging HCPs’ experiences with safety protocol and subsequent intentions to shift their practices can expand long-term capacity at the individual, clinic, and, ultimately, health system levels, rendering the intervention a worthwhile investment. Lastly, it is important to systematically engage HCPs in clinical decision-making components of the intervention, such as ART regimen changes that integrate resistance testing.

## Supporting information

S1 Interview guide Project YES! in-depth interview guide for health care providers. (DOCX)
