# Supplementary material for: “Adolescents do not only require ARVs and adherence counseling”: A qualitative investigation of health care provider experiences with an HIV youth peer mentoring program in Ndola, Zambia
Source: PLoS One. 2021 Jun 9;16(6):e0252349. doi: 10.1371/journal.pone.0252349 (PMC8189477; doi:10.1371/journal.pone.0252349)
Supplement: S1 Interview guide — (DOCX) [file pone.0252349.s001.docx]

**Project YES! In-depth Interview Guide for Health Care Providers**

Study Title: Transitioning Adolescents to HIV Self-Management in Ndola, Zambia

**U.S. Principal Investigator:** Dr. Julie Denison, Assistant Professor, Johns Hopkins Bloomberg School of Public Health

**Zambia Principal Investigator:** Dr. Jonathan K. Mwansa, Senior Medical Superintendent, Arthur Davison Children’s Hospital

**Study Sponsor**: USAID/Project SOAR/ADCH/Johns Hopkins Bloomberg School

of Public Health

**JHU IRB#:** IRB00007870

**Date of interview (DD/MM/YY): _________________**

**Participant ID: __________**

**Position: ______________**

**Select Clinic:  1  2  3  4**

**Interview start time: _______ Interview end time: _______**

**Interviewer Initials: _______**

***Introduction:*** Great, now we may start the interview. We are doing this work to help the clinic improve care for young people. We want to understand your experiences with implementing Project YES. This information is confidential. You can also refuse to answer any question. Please feel free to tell us anything about your experiences with the program – both good and bad. There are no “right” or “wrong” answers; we are here to learn from you. Let us start with some questions about the peer mentoring program.

***Instructions for Interviewer:*** *Turn on the recorder. Record your name, the participant’s ID, date, current time, and interview location (spoken into recorder).*

** Overall Program Experiences**

- Please share with me your role in the clinic.
- What was your understanding of the purpose of the Project YES peer-mentoring program? What was Project YES meant to do or achieve?
- What was your role in Project YES?
  - What were your responsibilities in this role?
- Please tell me about your experiences with the Project YES peer-mentoring program.
  - What did you like and dislike about the program?
- How do you think the program helped or did not help youth at this clinic?

** Experiences with peer mentors (from HCP perspective)**

- Tell me about your experiences with the peer mentors. (Probe on their role, their performance, and how helpful they were/weren’t)
- How have peer mentors fit in at the clinic? What additional oversight, if any, do you feel is needed for the peer mentors? Have you had space for the peer mentors to work with clients?
- How often did you meet with the youth peer mentors? Tell me about such a meeting.
- How can we utilize peer mentors to best support you as a healthcare provider?
- How was your experience with the initial orientation meetings with youth, peer mentors, and caregivers?
  - Were you able to attend these orientation meetings? What made attending easy/difficult?
  - What did you like/not like about these orientation meetings?
  - Please tell me about a typical orientation meeting – what happened? What were these meetings like?
- What was helpful/not helpful about these initial orientation meetings?

** Experiences with caregiver group sessions (from HCP perspective)**

- What were your experiences with the caregiver group meetings?
- How many did you facilitate? (Probe on what made attendance easy/hard and what happened if they missed a meeting)
- What made it easy/hard to facilitate these group discussions? What would have helped you feel more prepared to facilitate?
- How comfortable did you feel discussing sensitive issues with caregivers, like sex and alcohol?
- What do you think motivated the caregivers to attend these sessions?
- Would caregivers come if there was no transport reimbursement?
- What did you think of the group meeting outlines?
- What would you change about these caregiver group meetings? What would you keep the same?

** Logistics**

- What are your thoughts about where the group caregiver meetings were held? Do you feel like participants could speak freely? Why/why not?
- In the future, where would you recommend these meetings take place?

** Youth and transitioning to adult care**

- What has been your experience with youth transitioning to adult care in the past?
  - When do youth generally transition to adult clinics (if ever)?
- What has been your experience with the youth transition process as part of Project YES?
  - How did this process compare to other transition experiences you have had?
  - Tell me about a successful patient transition experience, and tell me about an unsuccessful experience.
- *Pediatric Clinic providers*: One of the suggested parts of Project YES was to have a Pediatric Clinic HCP attend the first clinic visits of transitioned clients at the Adult Clinic. – What was your experience with this (did it happen)?
  - If yes, what did you think of those meetings?
  - If no, do you think such meetings would help youth? What would make it easy/difficult for HCPs to attend those meetings?
- How could this transition process have been improved?

** Youth self-management**

- How have things stayed the same or changed for your youth patients who participated in Project YES! and met with a youth peer mentor? (probe on taking their drugs and attending their clinic appointments)
- What do you think are the key things that make it easy or hard for youth to take their medication and attend clinic appointments (probe for issues of alcohol use, violence, sexual experiences and mental health, stigma)
- How, if at all, has participating in the program changed the way you think about your patients?

** Experiences with patient drug changes**

- Some participants in this program showed resistance to their current ART drugs and were recommended to have their drugs changed. What was your experience with this process?
- What worked well/what didn’t work well?
- How can this process be improved?

** Referral Process Experiences**

- What were your experiences with the referral process in Project YES? (during data collection or program activities)
- Could you walk me through the steps taken for a referral? (probe on baseline and YPM referrals)
  - How were participants brought to you (probe on from whom – data collector or YPM)? How quickly were you able to see participants?
  - How did you feel about signing the referral forms? Completing the referral book?
- Tell me about a time when a participant was referred to you and it went well/it was difficult. Who referred them (Research Assistant or Youth Peer Mentor)?
- Are you still seeing any of the patients who were referred to you through Project YES for their referral issue?
- Have you referred any participants to another provider or organization for additional care? Please explain.
  - Where did you refer the participants? What were the reason(s) that you referred them for additional care? Did the participants meet with the person(s) you referred them to?
- Tell me about your interactions with our study staff on referrals.
  - Were you mostly satisfied with the support our staff provided? Mostly dissatisfied?
- How helpful do you think the referrals were for the youth client?
- What, if anything, would you change about the referral process in Project YES?

** Program Recommendations**

- Would you want this program to continue? Why/why not?
- Would you recommend this program to others? Why/why not?
- How did you feel about participating in a research study like this one?
  - Have you participated in other research studies?
- What can we do to make the program better?
- Is there anything else you would like us to know?
